# Hysteroscopic tissue removal systems for the treatment of intrauterine pathology: a systematic review and meta-analysis

**Published:** 2018-12

**Authors:** X Yin, J Cheng, SH Ansari, R Campo, W Di, W Li, G Bigatti

**Affiliations:** Sino European Life Expert Centre–Department of Obstetrics and Gynaecology, Renji Hospital, School of Medicine, Shanghai Jiao Tong University, Shanghai, China;; Shanghai Key Laboratory of Gynaecologic Oncology, China;; Department of Obstetrics and Gynaecology, Day General Hospital, Tehran, Iran;; Life Expert Centre, Leuven, Belgium.

**Keywords:** Myoma, Polyp, Truclear®, Myosure®, Hysteroscopic Shaver, System Review, Meta-analysis

## Abstract

**Background:**

The use of mechanical tissue removal systems is more frequently implemented as the first line approach for the treatment of intrauterine pathology. Scientific evidence is provided that their use is easier and faster than the conventional resectoscope. It is necessary to objectively evaluate the results on tissue removal systems for the treatment of endometrial pathology as the reports in the literature are still conflicting.

**Objective:**

To review and compare mechanical hysteroscopic tissue removal systems (Truclear®, Myosure® or IBS®) versus conventional bipolar and monopolar resectoscopy for the treatment of polyp and myoma removal. Operation time, completeness of tissue removal, complication rate, fluid deficit, tolerability and learning curve were evaluated.

**Methods:**

Electronic databases PubMed; Medline and Web of Science were searched for papers published from 1st January 2010 to 1st May 2019 using terms: (“hysteroscopic” or “hysteroscopy” or “hysteroscopic surgery”) and (“myoma” or “polyps”). Studies were included if they were retrospective, observational and prospective randomized clinical controlled trials if they investigated the techniques between the tissue removal systems (Truclear®, Myosure® or IBS®) and conventional resectoscopy for the treatment of intrauterine pathology. Data were extracted from the included studies by two independent reviewers. Meta-analysis was performed by Rev Man 5 software (Cochrane Collaboration, London, UK). Results: Overall, 498 patients were analysed from five studies in which there was no difference in age and size of pathology treated either by the hysteroscopic tissue removal systems and the conventional resectoscope. Hysteroscopic tissue removal systems showed a significantly higher success rate of complete endometrial pathology removal (P=0.002) and a significantly shorter operation time for polyp removal (P<0.0001) compared to conventional resectoscopy. No significant differences, in terms of complications rate, were found (P=0.09). The fluid deficit was significantly higher in the tissue removal system group, compared to conventional resectoscopy (P=0.02).

**Conclusion:**

Hysteroscopic tissue removal systems showed a major advantage in successful removal of the pathology and total operation time. It is likely that the tissue removal systems are more accessible and have a lower complication profile including perforation, via falsa and bleeding due to its specific action mechanism and shorter operation time but higher-quality trials will be required to confirm this hypothesis.

## Introduction

Abnormal uterine bleeding (AUB), is estimated to affect 30% of women in their fertile and postmenopausal age (Munro, 2014). Functional and structural disorders such as polyps and submucosal leiomyomas (AUB-P/L) are the main causes for abnormal uterine bleeding (Molinas and Campo, 2006; Campo et al., 2018). Conventional resectoscopy for the treatment of structural disorders has been used since 1970 with satisfactory outcomes (Jacobsen and DeCherney, 1997). Monopolar or bipolar current has been commonly used for pathological tissue resection. However, the risks of this procedure include cervical trauma, uterine perforation and fluid overload syndrome (Munro and Christianson, 2015). The risks are directly related with the experience of the surgeon and the size of the pathology. Although bipolar resectoscopy has reduced the risk of fluid overload syndrome, the resection time and removal of tissue chips from the uterine cavity are directly related with the size of the pathology and remains a time-consuming procedure. Frequent in and out movement or additional curettage is related with a risk of cervical laceration or uterine perforation.

With the recent introduction of new hysteroscopic instruments, a new approach to tissue removal was introduced. Hysteroscopic tissue removal systems with a modern fluid management system provides the possibility to remove large amounts of tissue in a simple and fast way. Many gynaecologists have reported the benefits of hysteroscopic tissue removal systems like shorter operation time, higher total resection rate, and higher patient acceptability (Meulenbroeks et al., 2017; Georgiou et al., 2018). Currently, there are three main tissue removal systems: Truclear®, Myosure® or IBS®. Most of the studies published investigated one of the tissue removal systems compared with conventional resectoscopy. There is little published data to evaluate and compare the three major tissue removal systems (Truclear®, Myosure® or IBS®) with conventional resectoscopy. Individual studies lack sufficient rigor to provide a precise evaluation. In addition, safety and efficacy of the tissue removal techniques have not been systematically evaluated by combining the data from all eligible studies. The purpose of our meta-analysis is to review and compare the different devices (Truclear®, Myosure® or IBS®), with conventional resectoscopy for the treatment of intrauterine polyp and myoma removal. Evaluation of operation time, completeness of tissue removal, fluid deficit and complication rate were included in the study.

## Materials and methods

As part of a systematic review and meta-analysis, electronic searches from three databases (Web of Science, PubMed and Medline), have been performed by two reviewers independently (XY and JC), to identify all relevant studies that evaluated hysteroscopic tissue removal system efficacy and safety for the treatment of endometrial polyps or myomas, compared with conventional resectoscopy. The search was limited to English language publications. The following search strategy was used for the literature search: (“hysteroscopic” or “hysteroscopy” or “hysteroscopic surgery”) AND (“myoma” or polyps”). The last search was conducted on May 1, 2019. Additional publications that had not been identified in the electronic searches were examined manually. Studies were included in the analysis if they were retrospective, observational and prospective randomized controlled trials and if the participants had endometrial lesions including endometrial polyps or submucous myomas, the intervention comprised of hysteroscopic tissue removal systems (Truclear®, Myosure® or IBS®) while the control group underwent conventional resectoscopy with monopolar or bipolar electrosurgical system, at least one of five outcomes were reported (success rate, fluid deficit, operation time, complications and complete removal). Studies were excluded if the identified publication described a non-comparative study or was a letter, case report, or review. Additionally, if the outcomes ‘data of interest’ were not described clearly, the study was also excluded. Authors of identified studies were contacted for clarification about methods and outcomes if necessary. Data was extracted from the included studies using a standard PRISMA data extraction form. General characteristics extracted included the author names, year of publication, country, number of patients and their age, hysteroscopic techniques used in the experimental and control groups, operative time, fluid deficit, complications and total removal rate. To ensure extracted data completeness and accuracy, two investigators (XY and JC) independently analysed the identified studies and then cross-checked their results. When differences arose between the two authors, the final decision was made through discussion. If the relevant data was not clear, the corresponding author of the original trial was contacted by email for missing or further information. The risk of bias was assessed by two independent authors (XY and JC) in accordance with the Cochrane Handbook for Systematic Reviews of Interventions. The following seven domains related to risk of bias were evaluated for each trial: (1) details of the randomization method, (2) concealment of treatment allocation, (3) masking of the participants and personnel, (4) blinding of the outcome assessment, (5) incomplete outcome data, (6) selective reporting, and (7) other biases. For each criterion, the risk of bias was rated as low, high, or unclear. Disagreement was resolved by discussion. Data synthesis was performed by two independent authors (XY and JC) using Rev Man 5.2 (Cochrane Collaboration, London, UK). Continuous data was presented as mean value and standard deviation, and the weighted mean difference and 95% confidence interval (CI) was calculated. Dichotomous data was expressed as odds ratios (ORs) with 95% CIs.

P<0.05 was considered statistically significant. The heterogeneity in outcomes across trials was assessed using the χ2 and I2 tests. An I2 value of more than 50% was considered to indicate substantial heterogeneity, which prompted use of a random-effects model for the analysis. Otherwise, a fixed-effects model was applied. Publication bias was assessed with a funnel plot in which the sample size was plotted on the y-axis, and the pooled OR for the complete removal of endometrial lesions on the x-axis. Bias was considered to be present if the plot was asymmetrical, whereas a plot resembling a symmetric funnel showed that there was no bias.

## Results

### Literature search

**Figure 1:**
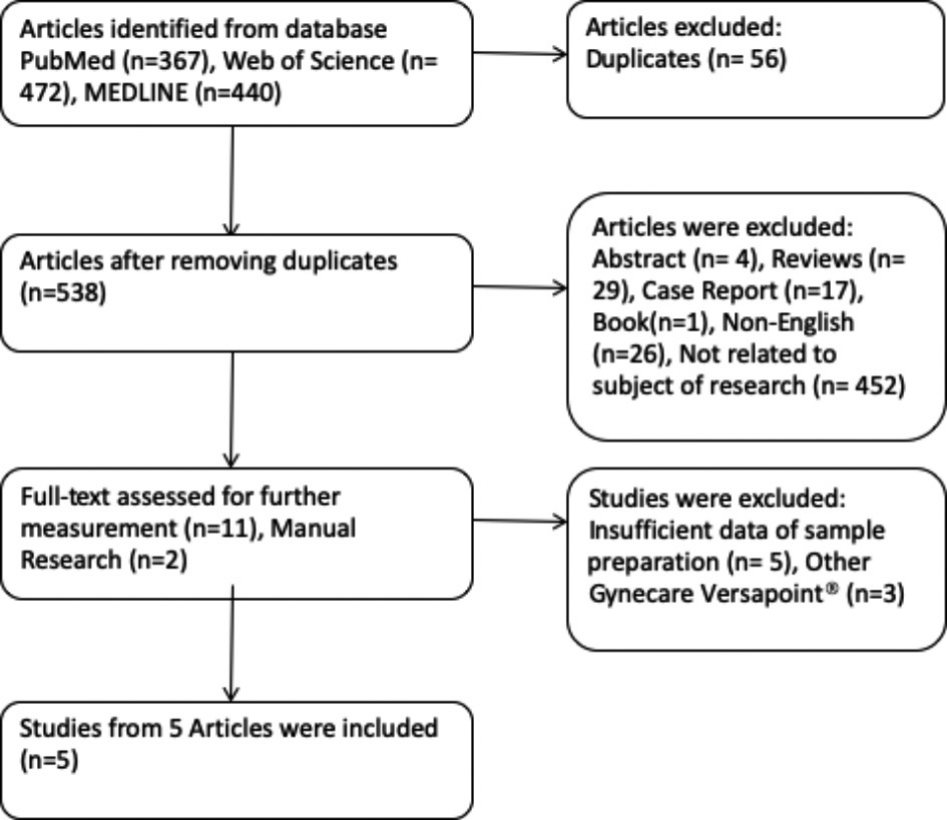
Flow diagram of eligible studies selection process.

A total of 538 reports were identified on the basis of the predefined search strategy (Figure 1). 13 reports underwent full-text review after which, 5 studies were excluded because of insufficient sample data preparation (n=5), and 3 studies were excluded because the control groups for Versapoint® did not refer to conventional resectoscopy, but to the bipolar probe use.

### Main characteristics of included studies

Five studies with a total of 498 patients were included for further data extraction and meta-analysis (Bigatti et al., 2012; 2014; Hamerlynck et al., 2015; Lee and Matsuzono, 2016; Tsuchiya et al., 2018). Overall, 215 patients were included in study groups and 183 patients in control groups. All selected reports were published in English. All procedures in all studies of the study groups were performed with hysteroscopic tissue removal systems (Truclear®, Myosure® or IBS®), while the control groups were operated by conventional resectoscopy. For the conventional resectoscopy procedure, two studies from Bigatti et al. (2012; 2014) used 26 Fr bipolar electrosurgical systems (Karl Storz SE & Co KG), one study (Hamerlynck et al., 2015) used the 8.5 mm bipolar resectoscope (Gynecare Versapoint®), and the 8.6 mm conventional monopolar resectoscope was used by Lee et al. (2016) and Tsuchiya et al. (2018) (Olympus Corp). Three studies reported endometrial myomas as primary lesion, one study included polyps and myomas, whereas the last study reported only endometrial polyps. A detailed description regarding the characteristics of the five studies is reported in Table I.

**Table I:** Spermatozoa mean binding to hemi-zonae after insemination of sperm (95% CI, n=104).

Study Type	Inclusion criteria	Exclusion criteria	Age, y^b^	No. of patients	Surgical technique		(95% CI)
					Experimental	Control	
Bigatti et al., 2012	Randomized comparative	Polyps as large as 6 cm, and G0, G1 and G2 submucosal myomas (classified according to the ESGE guidelines) that were up to 3 cm in diameter	Uterine malformations such as partial or complete septum ablations an oncological cases	48.6	95	24 Fr IBS®	Bipolar resectoscope 26 Fr (Karl Storz SE & Co KG)	General anaesthesia
Bigatti et al., 2014	Retrospective comparative	Operative hysteroscopy of myomectomies, and polypectomies with the IBS® versus women with the Versapoint® over a 2-year period, from June 2011 to June 2013, in Ospedale San Giuseppe of Milan-Italy	Oncological cases	47.6	127	24 Fr IBS®	Bipolar resectoscope 26 Fr (Karl Storz SE & Co KG)	General or regional anaesthesia
Hamerlynck et al., 2015	Randomised comparative	At least 1 large (≥ 1 cm) endometrial polyp, and hysteroscopic removal was needed	Visual or pathological evidence of malignancy, untreated cervical stenosis, or the presence of a contraindication for operative hysteroscopy	50.5	84	Trueclear 8.0	Bipolar resectoscope 8.5 mm (Gynecare Versapoint®)	General anaesthesia and spinal anaesthesia
Lee et al., 2016	Retrospective comparative	Hysteroscopic resection of submucosal fibroids were performed at Queen Elizabeth Hospital , Hong Kong, between 1st of January 2011 and 31 st of December 2014, either by IUM (Myosure) or conventional hystescopic monopolar loop resection	Those cases with prolonged operating time due to multiple operations for other indications or complications were excluded	NR	25	Myosure	Monopolar Resectoscope	General anaesthesia
Tsuchiya et al., 2018	Randomised comparative	All eligible women were diagnosed as having endometrial polyps based on an office hysteroscopy	(1) patients who received hysteroscopic polypectomy before, (2) patients with uterus bipartus, (3) patients with intrauterine adhesion, and (4) patients with endometrial carcinoma or suspected of having endometrial carcinoma	38.3	67	Trueclear 8.0	Monopolar resecto-scope 8.6 mm (Olympus Corp.)	General anaesthesia

### Quality assessment

**Figure 2:**
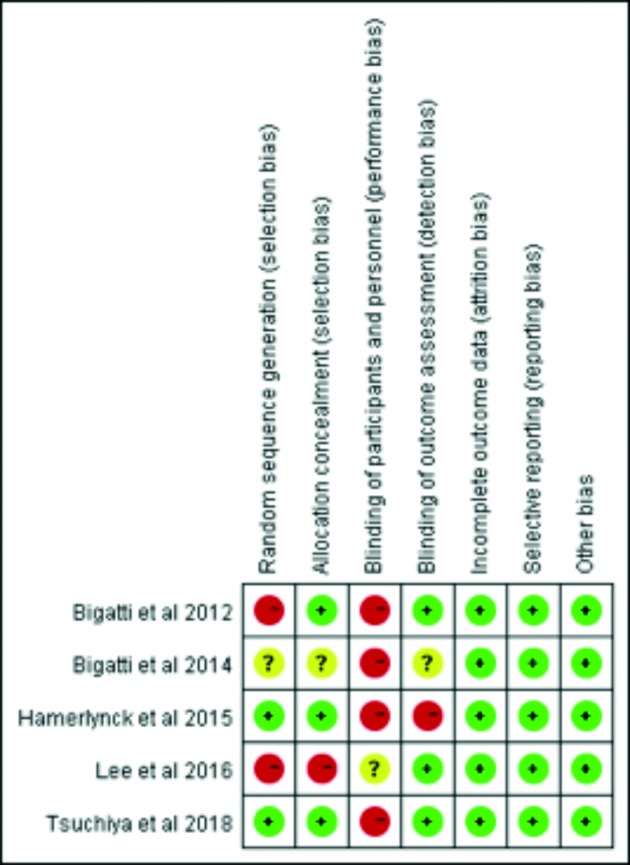
Risk of bias summary: review authors’ judgements about each risk of bias item for each included study.

The Cochrane’s tool was employed for the risk of bias appraisal, and for the included randomized control trials evaluation. All the studies, except Bigatti et al. (2014) claimed randomization. Three studies failed to report allocation concealment. In the included studies the blindfolding of participants and surgeons was impossible, therefore the performance of bias was not reported. One study failed to report the operative outcome of blinding assessment and it went to unclear risk of bias. Exclusion was applied when incomplete outcome data was reported. One study was judged unclear because no statistical analysis regarding total removal rate was performed (Lee et al., 2016). The reviewers’ judgments about each risk of bias is described in Figure 2. Although no large sample size (25–227 participants) was reported, each one in the included study was well designed. In the included studies there was no bias for incomplete or selective outcome reporting.

### Difference of age in both groups

Four studies reported detailed data regarding the age of patients, and there was no significant difference between the tissue removal system group and the conventional resectoscopy group (P=0.13). The data showed that the general conditions in the two studied groups were similar.

### Difference of pathological size in both groups

As to the size of submucosal myoma and polyp, the result showed that there was no difference between the experimental and the control group (P=0.85). The mean size of endometrial pathology ranges from 16 to 23.12 mm in tissue removal system group and 9.67 to 25.18 mm in conventional resectoscopy group.

### Difference of operation time in both groups

Five studies, reporting the total operation time taken for complete removal of endometrial pathology, were pooled. The results showed that hysteroscopic tissue removal system was not faster than conventional resectoscopy, (mean difference −0.33 minutes, P=0.83). Among them, operation time for only polypectomy, was evaluated by three studies. Polypectomy time proved to be significantly shorter with tissue removal system than with conventional resectoscopy (mean difference -2.93min, 96%CI: -4.25, -1.61, P<0.0001). No difference for myomas complete removal time was reported (P=0.46).

### Difference of total removal rate in both groups

Three studies investigated total removal rate of endometrial pathology. The pooled results showed that the success rate was higher with hysteroscopic tissue removal system than with conventional resectoscopy, (147/154 [95.5%] vs 107/124 [86.3%]; OR 4.28, 95% CI: 1.68–10.91; P=0.002). There was no significant heterogeneity among the included trials (I2=0%, P=0.83). Our analysis results showed that tissue removal systems were more effective than conventional resectoscopy in removing endometrial pathology.

### Difference of fluid deficit in both groups

All five studies showed that the tissue removal systems fluid deficit was higher than conventional resectoscopy (mean difference 158.98 mL, 95% CI: 41.42 to 276.54; P=0.008), but no fluid overload syndrome was reported. Fluid deficit was significantly higher for myomectomy treated with tissue removal systems (P=0.02). However, this data was not confirmed for polypectomy (P=0.88).

### Difference of complications in both groups

Four studies reported intraoperative and postoperative complication rates, including perforation, via falsa and bleeding. No significant difference between hysteroscopic tissue removal systems and conventional resectoscopy (OR 0.31, 95% CI: 0.08–1.19; P=0.09) was reported. No significant difference, in terms of complication rate concerning polypectomy, was observed in a subgroup comparing hysteroscopic tissue removal systems with conventional resectoscopy (P=0.13).

## Discussion

To our knowledge, this is the first systematic review and meta-analysis comparing the use of the three most important tissue removal devices (Truclear®, Myosure® or IBS®) with the conventional mono and bipolar resectoscope for the treatment of intrauterine polyps and submucous myoma.

In this meta-analysis, strict criteria were used to select 5 scientific publications from the original 538 papers. A total of 498 patients from 5 studies, 215 in the experimental group and 183 patients in the control group, were included. There has been one published meta-analysis by comparing hysteroscopic tissue removal with resectoscopy for patients with endometrial lesions (Li et al., 2013). Li et al. (2013) reported four published articles: van Dongen et al. (2008), Smith et al. (2014), Pampalona et al. (2015) and Hamerlynck et al. (2015. However, because of different selection criteria, only one publication was included in our analysis. Overall, the quality of all these 5 publications was considered good with moderate bias.

From the present study, the pooled meta-analysis indicated that the age of participants was not significantly different between hysteroscopic tissue removal system and the conventional resectoscopy group, with a heterogeneity of 0% and P value of 0.13. The average age of all five publications ranges from 37 to 51 years old, which is consistent with intrauterine pathology prevalence (Anastasiadis et al., 2000). The size of endometrial lesions is strongly related to operation time, complications and pregnancy rate (Stamatellos et al., 2008). From our meta-analysis results, the pooled sizes showed no significant difference between hysteroscopic tissue removal systems and conventional resectoscopy, with a heterogeneity I2 of 55% and P value of 0.85. Although heterogeneity is moderate, polyps and myomas have different histological properties and should be discussed separately. These results represent a prerequisite condition of further analysis between hysteroscopic tissue removal systems and conventional resectoscopy, as well as between polyps and myomas.

Operation time and total removal rate are parameters strictly related to the success outcome of operative hysteroscopy. In our meta-analysis of polypectomy but not of myomectomy operative time, reveal a significant difference for hysteroscopic tissue removal systems, compared to conventional resectoscopy (P<0.0001 and I2=0%). A mean reduction time of 2.93 minutes is observed for hysteroscopic removal systems. Here the time is restricted to operation time including installation time and insertion time, regardless of anaesthesia time (Hamerlynck et al., 2015; Tsuchiya et al., 2018). In the study of Bigatti et al. (2012) the time for the procedure, without the dilatation time, was regarded as the total operative time. Steam bubbles produced by electrically heated saline solution and the removal of the resected tissue fragments, impair visibility inside the uterine cavity.

Our meta-analysis shows that hysteroscopic tissue removal systems (Truclear®, Myosure® or IBS®), are more successful in completely removing endometrial lesions than conventional resectoscopy. The advantage of this new hysteroscopic resection technique is defined by the rate of complete removal of pathological tissue. The present pooled results indicate a higher success rate for patients treated with a hysteroscopic removal system (95.5%), than with conventional resectoscopy (86.3%). The major reasons for a one-step procedure lower level of success in conventional resectoscopy is related to the fluid deficit and to the operation time limitations. Bigatti et al. (2014) reported that a procedure had to be stopped, even if resection was incomplete, when a limit of 2000 ml fluid deficit or if an hour-long whole procedure time was reached, in order to avoid fluid overload syndrome (Bigatti et al., 2014). Although one subgroup analysis indicated that the shaver technique contributed to treating 93.5 % of myomas with a diameter less than 3cm, the operation success rate was affected by the size rather than the myoma type. In a prospective multicentre trial where the patients underwent hysteroscopic mechanical tissue removal of uterine polyps and myomas, Scheiber and Chen (2016) found similar results with an overall mean percentage of pathology removed of 99.3% for polyps and 86.8% for fibroids.

With a much faster operation time, less fluid deficit was expected. However, our pooled meta-analysis has shown a much higher fluid deficit in the hysteroscopic tissue removal systems group, compared to conventional resectoscopy. One possible explanation could be the fast and high-pressure fluid pumping device used by Lee et al. (2016) in order to maintain a clear view, and to prevent bleeding inside the uterine cavity. Although hysteroscopic tissue removal systems have shown a much higher fluid deficit, no overload syndrome in our pooled data was reported (Isaacson and Olive, 1999).

Furthermore, complications such as perforation, via falsa, bleeding at follow-up, and the need for additional treatment, have been analysed. Hysteroscopic tissue removal systems show a low complications rate, with no significant difference between the two groups (P=0.09) reported. No significant difference was observed, in terms of complication rate, concerning polypectomy in a subgroup comparing hysteroscopic tissue removal systems with conventional resectoscopy (P=0.13). Al Hilli et al. (2013) showed a very low complication and recurrence rate of polyps when treated with intrauterine morcellation systems. Hamerlynck et al. (2011) performed a Truclear retrospective study on 315 women. As no complication was reported, they came to the conclusion that this morcellation system was a very safe method to treat polyps and small submucosal myomas, but unfortunately no comparable control group was available. Further data is needed in order to confirm these results. Post-surgical adhesion formation after operative hysteroscopy is another important issue. Deans and Abbott reported adhesions de novo formation, after the removal of respectively single and multiple myomas, in 31.3 % and 45.5 % of cases (Deans and Abbott, 2010). In only one of our selected studies, no occurrence of intrauterine adhesions formation at second look hysteroscopy was reported (Hamerlynck et al., 2015). Additional data should be collected to reach a reliable conclusion.

Potential limitations of the present meta-analysis should be taken in consideration. Firstly, the number of included studies is limited. Only five studies have been observed and the sample sizes are relatively small, raising the risk of a possible bias of analysis. It is recommended that additional randomized control trials, with larger sample size and different cultural contexts, take place. Pooling the data on recurrence rate and learning curve is impossible because of inclusion criteria and outcome parameters differences among the studies. Lastly, no study reports cost-effectiveness evaluation of a hysteroscopic tissue removal system.

## Conclusions

As operative hysteroscopy is fast becoming the first-choice procedure for treating intrauterine pathology, it is becoming mandatory to find a more effective technique. Based on the available evidence, the present meta-analysis shows that hysteroscopic tissue removal systems (Truclear®, Myosure® or IBS®) provide a very fast, precise and safe alternative to conventional resectoscopy. Our preliminary results demonstrate that hysteroscopic removal systems hold great potential to replace conventional resectoscopy for the treatment of polyps and less than 3 cm myomas. Although potential benefits of hysteroscopic tissue removal systems have been clearly shown in this paper, other possible surgical indications like large myomas, uterine septa, placental remnants, intrauterine adhesions with infertility treatments, should be investigated by additional clinical trials. Other aspects such as cost effectiveness and learning curve should be also evaluated. Finally, a comparison among different hysteroscopic tissue removal systems should be performed in order to describe advantages and possible drawbacks of the different devices.

## Declaration of Interests

Dr. G. Bigatti is a consultant for Karl Storz SE & Co KG and the developer of the IBS®.

Dr. R. Campo is a consultant for Karl Storz SE & Co KG.

No financial industry support was received for this study.
